# Effects of bilateral anterior agranular insula lesions on food anticipatory activity in rats

**DOI:** 10.1371/journal.pone.0179370

**Published:** 2017-06-08

**Authors:** Alex M. Gavrila, Suzanne Hood, Barry Robinson, Shimon Amir

**Affiliations:** 1 Department of Psychology, Center for Studies in Behavioural Neurobiology/FRSQ Groupe de Recherche en Neurobiologie Comportementale, Concordia University, Montreal, Quebec, Canada; 2 Department of Psychology, Bishop's University, Sherbrooke, Quebec, Canada; Kent State University, UNITED STATES

## Abstract

Food anticipatory activity (FAA) refers to a daily rhythm of locomotor activity that emerges under conditions of food restriction, whereby animals develop an intense, predictable period of activity in the few hours leading up to a predictable, daily delivery of food. The neural mechanisms by which FAA is regulated are not yet fully understood. Although a number of brain regions appear to be involved in regulating the development and expression of FAA, there is little evidence to date concerning the role of the anterior agranular insular cortex (AICa). The AICa plays a critical role in integrating the perception of visceral states with motivational behaviour such as feeding. We assessed the effect of bilateral electrolytic or ibotenic acid lesions of the AICa on FAA in male Wistar rats receiving food for varying lengths of time (2 h, 3 h, or 5 h) during the middle of the light phase (starting at either ZT4 or ZT6). Contrary to our initial expectations, we found that both electrolytic and ibotenic acid lesions significantly increased, rather than decreased, the amount of FAA expressed in lesioned rats. Despite increased FAA, lesioned rats did not eat significantly more during restricted feeding (RF) periods than control rats. Similar to controls, AlCa-lesioned rats showed negligible anticipatory activity to a restricted treat suggesting that the increased anticipatory activity in lesioned rats is associated with food restriction, rather than the appetitive value of the meal. Monitoring behaviour in an open field indicated that increased FAA in AlCa-lesioned rats was not explained by a general increase in locomotor activity. Together, these findings suggest that the AICa contributes to the network of brain regions involved in FAA.

## Introduction

Feeding schedules that restrict food availability to a fixed time of day induce and entrain food anticipatory rhythms, which are marked by a daily increase in arousal and locomotor activity in the 2-to-4 h period prior to the scheduled meal (reviewed in [1]) commonly referred to as food anticipatory activity (FAA). Entrainment of food anticipatory rhythms is distinct and independent from entrainment of circadian locomotor activity rhythms by the light-dark cycle, which is mediated by the suprachiasmatic nucleus (SCN), the master circadian clock [2,3]. Evidence suggests that multiple brain regions are involved in FAA, although the specific mechanisms underlying food entrainment remain unclear. Lesion studies aimed at identifying candidate brain areas have focused on structures important in feeding, motivation, and locomotion. These include the ventromedial hypothalamic nucleus [4–7], dorsomedial hypothalamic nucleus [8], lateral hypothalamus [9], paraventricular hypothalamic nucleus [9,10], thalamic paraventricular nucleus [11], hippocampus [12], amygdala [12], striatum [13], infralimbic cortex [14], parabrachial nucleus [15], nucleus of the solitary tract [16], area postrema [17], cerebellum [18], and olfactory bulbs [19]. Although damage in select structures, notably the parabrachial nucleus, dorsomedial hypothalamic nucleus, infralimbic cortex, and cerebellum led to deficits in some measures of FAA, none of these areas alone was found to be necessary for the timing or expression of all food anticipatory circadian rhythms. This suggests that these rhythms are regulated by a distributed brain network of food-entrainable clocks, and not by a central clock structure.

The insular cortex plays a key role in integrating visceral and somatic signals, and influencing goal-directed behaviours with this information [20]. The insular cortex is divided into three main sub-regions: granular, dysgranular, and anterior agranular insular cortex (AICa). Projection studies have shown that, apart from having reciprocal connections with one another, each sub-division also has distinct reciprocal connections with specific brain regions [21,22]. The granular and dysgranular insular cortices are primarily interconnected with visceral thalamic relay nuclei and are therefore important in monitoring and integrating interoceptive stimuli [22]. The AICa has numerous afferent and efferent projections to key limbic structures such as the nucleus accumbens, medial prefrontal cortex, and the amygdala [23], which suggests that the AICa potentially plays an important role in regulating motivated behaviours. Furthermore, the insular cortex appears to contribute to the processing of gustatory signals [24]. For instance, as the AICa is adjacent to and highly interconnected with the primary taste cortex and receives gustatory afferents from the parabrachial nucleus [25]. Given these roles of the AICa, we hypothesized that it may be important for supporting food entrainment, and that its inactivation would negatively impact the amount of FAA observed. Specifically, we expected to find that bilateral lesions of the AICa of Wistar rats would significantly decrease FAA. Instead, we found that both electrolytic and ibotenic acid lesions produced a significant increase in FAA, especially before a 2 h meal.

## Materials and methods

### Animals and housing

Sixty-four adult male Wistar rats (Charles River Laboratories, St-Constant, QC, Canada) weighing between 240–275 g at the start of each experiment were used. Each rat was housed individually in a clear plastic shoebox cage (24 × 20 × 37 cm) equipped with a running wheel throughout the experiment. Each cage was individually housed within a sound- and light-proof custom-made cabinet (66 × 66 × 44 cm) equipped with a computer-controlled fluorescent light source (approximately 300 lux at cage level) and a ventilation system. Running-wheel activity was recorded continuously and displayed in 10 min bins using VitalView software (MiniMitter Co., Sunriver, OR, USA). The rooms in which the rats were housed were maintained at 21°C throughout the testing period. All procedures were in accordance with the Canadian Council on Animal Care guidelines (http://www.ccac.ca) and were approved by the Animal Care Committee at Concordia University (Montreal, QC, Canada). Rats had *ad libitum* access to standard rodent diet (#5075, Charles River Laboratories, St-Constant, QC, Canada) and water during all experiments, except during bouts of restricted feeding when only water was provided *ad libitum*.

### Experimental design

**Fig 1:**
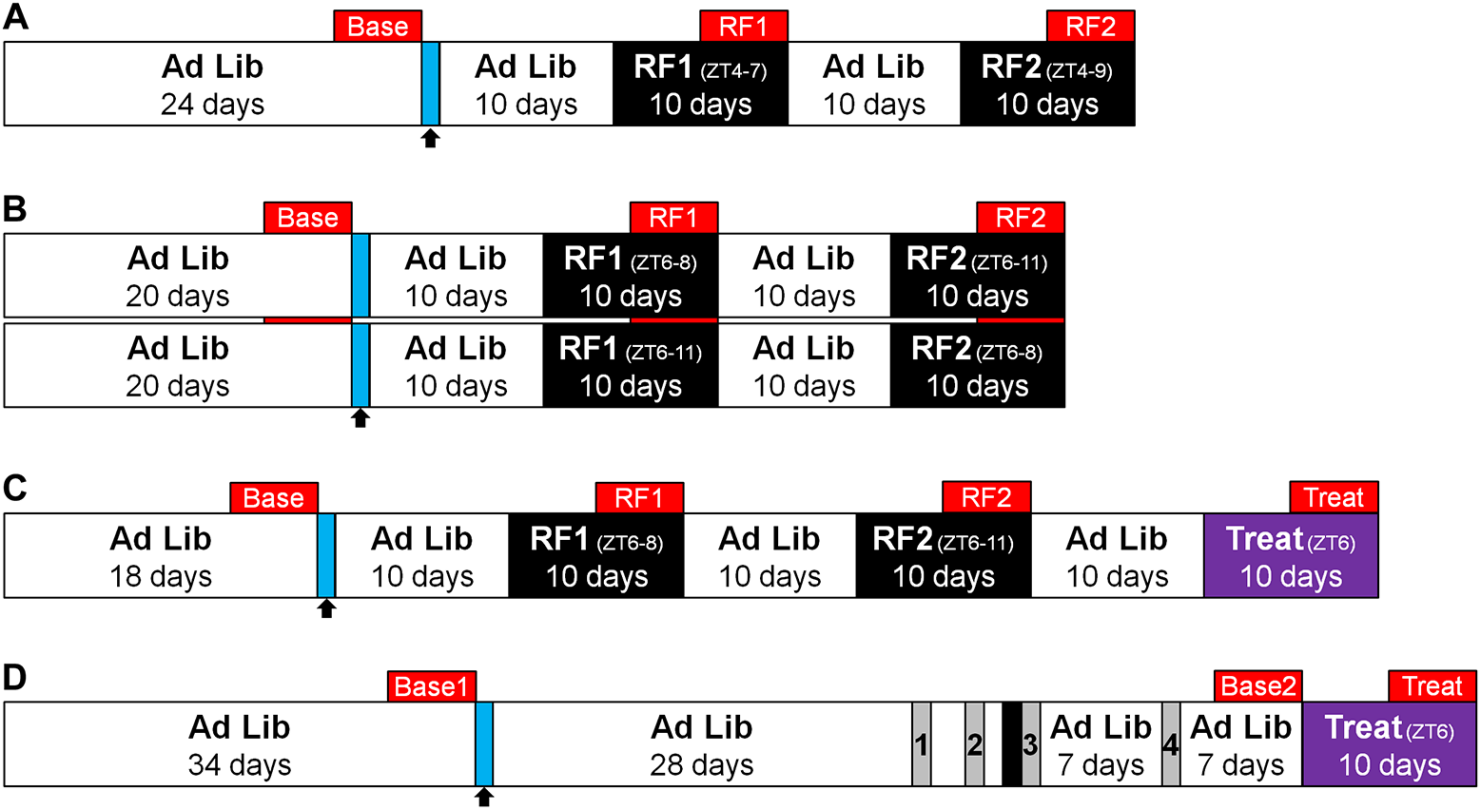
Experimental overview. Red boxes indicate the 5-day periods for which data are presented. Arrows and blue lines indicate the days of surgery. (A) Outline of experiment 1. (B) Outline of the counter-balanced feeding schedule for experiment 2. (C) Outline of experiment 3, including the two bouts of restricted feeding (RF) and the 15 min/day of treat while regular rat chow was available *ad libitum*. (D) Outline of experiment 4. Numbers 1–4 indicate periods when rats were placed in an open field for 1 h. All rats were food deprived for 24 h (red bar) prior to the third open field exposure in order to ascertain activity levels following acute food deprivation. Lastly, a chocolate Ensure treat was provided for 15 min starting at ZT6 for 10 days.

Four experiments were carried out to evaluate the role of the AICa in FAA. Fig 1 shows an overview of each experimental design.

#### Experiment 1: Effect of bilateral electrolytic AICa lesions on FAA

In experiment 1 (Fig 1A) we ascertained the effects of bilateral electrolytic lesions of the AICa on FAA in rats that had access to food for only 3 h (restricted feeding period 1, (RF1)) or 5 h (RF2) starting at Zeitgeber time (ZT4; 4 hours after lights were turned on). We expected that bilateral electrolytic lesions of the AICa would lower overall FAA, as the rewarding aspects of food and the interoceptive awareness of food deprivation would be disrupted.

#### Experiment 2: Length of food availability and FAA

In experiment 2 (Fig 1B) we further investigated the effects of bilateral electrolytic lesions of the AICa on FAA in rats under a 2 h (RF1) or 5 h (RF2) schedule starting at ZT6. Additionally, any potential carryover effects on activity levels and weight from RF1 to RF2 were assessed by counter-balancing the length of food access for half of the experimental and half of the control rats (refer to Fig 1B for graphical representation and to the appropriate methods sub-sections for complete details on the procedure followed).

#### Experiment 3: IBO AICa lesions and restricted treat

For experiment 3 (Fig 1C) ibotenic acid (IBO) was chosen as an additional means of lesioning the AICa, due to its cytotoxic properties. While electrolytic lesions result in physical damage of the targeted area, destroying both local cells and distant projecting fibers, ibotenic acid affects primarily local cell bodies, thus creating a more selective lesion [26]. Furthermore, as a number of recent studies have shown differential effects of restricted access to palatable treat given during *ad libitum* conditions and restricted feeding on FAA [27,28], the effects of AICa lesions on FAA during administration of a treat (chocolate Ensure) for 15 min at ZT6 in *ad libitum* fed rats during 10 consecutive days was also investigated. Lastly, bilateral dorsal control lesions were performed on a second control group to verify that the effects observed were due to lesioning the AICa and not the somatosensory cortical area dorsal to it.

#### Experiment 4: Effect of IBO lesions of the AICa on open field activity

Experiment 4 (Fig 1D) assessed the effect of bilateral IBO lesions of the AICa on overall activity and exploratory behavior in a novel environment (an open field). Four distinct variables were measured throughout each trial with a temporal resolution of 5 min: total number of movements, center entries, distance traveled, and number of rearings.

Experiment 4 used a TruScan 2.0 (Coulbourn Instruments, Whitehall, PA, USA) open field arena (42 × 42 × 30 cm) equipped with 2 super-imposed levels of motion sensors (photo-beams in each sensor ring were spaced 1.27 cm apart) to track each rat’s movements. Movement was tracked throughout each open field session, and motion maps were generated for each rat. The floor and all 4 walls of the open field were cleaned with ethanol (90% ETOH) before and after each rat to reduce distracting odors. Rats were placed in the open field for 1 h (from ZT6-ZT7) in order to determine whether any differences in activity levels would be seen between Sham rats (see surgery section below) and IBO rats in this novel environment. Two days later, the same procedure was repeated once more. The first exposure was used to ascertain the amount of activity exhibited in a novel environment, while the second was meant to serve as the baseline measure for subsequent open field trials. One day later, all rats were food-deprived for 24 h after which they were once again placed in the open field for 1 h to investigate the effect of acute food deprivation on activity levels of both Sham and IBO rats in an open field. Rats were placed in the open field for a fourth time 7 days later, to ascertain whether baseline behavior had been altered by the prior acute food deprivation.

### Surgery

All rats were housed in a 12–12 h light-dark cycle for at least 18 days before surgery to ensure stable photic entrainment and to provide an accurate measure of baseline wheel running levels. Zeitgeber time 0 (ZT0) was designated as the start of the lights-on period, and ZT12 as the start of the lights-off period. Rats were randomly divided into the following groups: an unoperated control group (CTRL), a dorsal control group (DCTRL), a sham-operated group (Sham), an electrolytic group (Electro), and an ibotenic acid group (IBO). All rats except those in the CTRL group were deeply anesthetized with ketamine/xylazine solution (100 mg/mL ketamine: 20 mg/mL xylazine; intraperitoneal injection 0.15 mL/100 g). Rats were securely fitted onto a stereotaxic apparatus and two small holes were drilled in the skull at the following coordinates (reference from bregma) for the AICa: anterior: +1.56 mm; and lateral: ± 5.0 mm. For the Sham and Electro groups, a bipolar electrode insulated except for the tip was mounted on a stereotaxic arm and slowly lowered to -6.2 mm below the skull. For Sham rats, the electrode was maintained in place for 15 s and then removed without passing any current through it. This procedure was followed in both the left and right hemispheres. For Electro rats, the electrode was lowered in one hemisphere and a 2 mAmp current was passed for 15 s. This procedure was then repeated for the other hemisphere. For DCTRL rats, an uninsulated electrode was lowered -3.0 mm below the skull and a 2 mAmp current was passed through it for 15 s, resulting in a large lesion covering the length of the electrode.

Rats in the IBO group received an infusion of ibotenic acid to create an excitotoxic lesion in the AICa. For excitotoxic lesions, a 10.0 μL Hamilton microsyringe was filled with ibotenic acid (2.5 μg/μL in saline; Baxter, Mississauga, ON, Canada) and mounted on a microinfusion pump (Harvard Apparatus, Holliston, MA, USA). Tubing was used to connect the syringe to a specially-designed injector tip, which was mounted on a stereotaxic arm. After slowly lowering the needle to -6.2 mm below the skull’s surface, the microinfusion pump was set to deliver a total of 1.0 μL ibotenic acid solution (2.5 μg) at a rate of 0.2 μL/min. Following the completion of the infusion, the injector was kept in place for an additional 1 min to allow the ibotenic acid to dissipate from the tip, then the contralateral side was lesioned following the same procedure. The holes in the skull were filled with sterile bone wax (Harvard Apparatus, Holliston, MA, USA) and subsequently covered with a layer of sticky wax. Hibitane (Bioglan, Sweden), an antifungal and antiseptic cream, was applied locally in order to prevent infection and the skin was sutured. Postoperative treatment comprised an injection of an antibiotic and analgesic (Procilin, 0.075 mL intramuscular injection in each hind leg), and fluids (Lactated Ringer’s solution, 3.0 mL subcutaneous injection).

### Restricted feeding regimen

Fig 1 shows the detailed timeline of each experiment. Rats were allowed to recover for at least 10 days after surgery. Immediately preceding the first bout of restricted feeding (RF1), all rats were food deprived for 24 h. Following this deprivation, rats received 35 g of food in their home cage for a fixed amount of time over 10 days (2, 3, or 5 h per day; refer to Fig 1 for detailed procedure for each experiment). Following RF1, rats were given *ad libitum* food for 10 days. A second 24 h period of food deprivation was then initiated, followed by a second 10-day bout of restricted feeding (RF2). The time of day at which rats received food during each RF period differed between experiment 1 and the other experiments. Specifically, in experiment 1 rats received food from ZT4-ZT7 during RF1 and from ZT4-ZT9 during RF2. For all other experiments, food was given at ZT6 for either 2 or 5 h. This change was made because it was determined that by shifting food presentation further into the light cycle by an additional 2 hours, it was easier to distinguish early anticipatory activity from any late nocturnal activity that may be present. Food consumption during RF1 and RF2 was recorded by weighing the leftover food at the end of each day. Rats were weighed every 2–5 days throughout each experiment.

### Histology

Rats were deeply anesthetized with an overdose of sodium pentobarbital (≈ 100 mg/kg, intraperitoneal injection: Somnotol, MTC Bimeda, Cambridge, ON, Canada) and perfused intra-cardially with 300 mL of cold saline (0.9% NaCl), followed by 300 mL of cold 4.0% formaldehyde. Brains were extracted and fixed for 24 h in 4% formaldehyde at 4°C. The following day, brains were sliced in cold phosphate buffered saline using a vibratome into two alternating series of ≈ 65 coronal sections (each 50 μM thick) and placed in cold trizma buffered saline. After three 10 min trizma buffered saline rinses to remove any remaining traces of formaldehyde, sections were transferred into Watson’s cryoprotectant [29] and maintained at—20°C. One of the two series of sections was used to assess the extent of each lesion.

Brain sections were inspected under a light microscope (Leitz Laborlux S, Germany) and images of the AICa were captured using a Sony XC-77 video camera connected to a Scion LG-3 frame grabber using NIH Image software (v1.63; http://rsb.info.nih.gov/nih-image/). Bilateral images were captured at 5×, 10×, and 20× magnifications using a 400 × 400 pixel template. Detailed lesion maps were subsequently sketched for each brain and only rats having clear bilateral lesions of the AICa were used for further analysis.

### Data analysis

#### Behavior

Single-plotted actograms were generated with ActiView (v1.2; MiniMitter Co.) and ActiWatch (v3.3; MiniMitter Co.), and were visually analyzed in order to ascertain entrainment and locomotor activity. For each 5-day period of data collection, a mean of the number of wheel revolutions was calculated for each 10-min bin of data (144 bins per 24 h). Total activity for the 12-h nighttime period and anticipatory activity (defined as the 3 h period immediately preceding food presentation) were selected for further analysis in SPSS (v13; Apache Software Foundation, DE, USA) and GraphPad Prism (v4.03; San Diego, CA, USA). For the analysis, the 5-day period prior to surgery was labeled as the Baseline, the last 5 days of the first bout of restricted feeding was labeled as RF1, and the last 5 days of the second bout of restricted feeding was labeled as RF2. These periods were selected because they showed the most consistent activity rhythms. Given idiosyncratic differences in endogenous circadian period length and sensitivity to food restriction, significant intragroup variance among rats during the first few days of RF can be often noted as some initially respond more readily than others, making comparison between groups difficult for the first few days of most experimental conditions. The last 5-day period of chocolate Ensure (Treat) administration in experiments 3 and 4 was also analyzed further.

#### Statistical analyses

Running wheel data from the CTRL and Sham groups were pooled where applicable because no differences were noted between these two groups in any of the measures used. Two-way mixed analyses of variance (ANOVAs) were run on the total wheel running activity for the 3 h anticipatory period prior to food presentation and total nighttime activity. Each ANOVA used experimental phase (Baseline, RF1, RF2, and Treat) as the within-subjects factor and group (CTRL/Sham, Electro, IBO, or DCTRL) as the between-subjects factor where applicable.

Changes in body weight were analyzed using a two-way mixed ANOVA with experimental days as the within-subjects factor and group as the between-subjects factor. Similar two-way mixed ANOVAs were run on food and Ensure consumption. Lastly, two-way mixed ANOVAs were used to monitor activity levels of rats placed in the open field. Specifically, the four dependent variables investigated were: Total number of moves, center entries, distance traveled, and number of rearings. For each of these dependent variables, the trial number (1 to 4) was used as the within-subjects factor and group (Sham or IBO) as the between-subjects factor. Mauchly’s test [30] and Levene’s test [31] were carried out in order to ascertain sphericity and homogeneity of variances. In some cases, when Mauchly’s test was significant, the Greenhouse-Geisser correction [32] was used in order to adjust the degrees of freedom. Planned comparisons or Bonferroni post hoc tests were conducted where applicable. All analyses used a significance level of α = .05.

## Results

### Experiment 1: Effect of bilateral electrolytic AICa lesions on FAA

**Fig 2:**
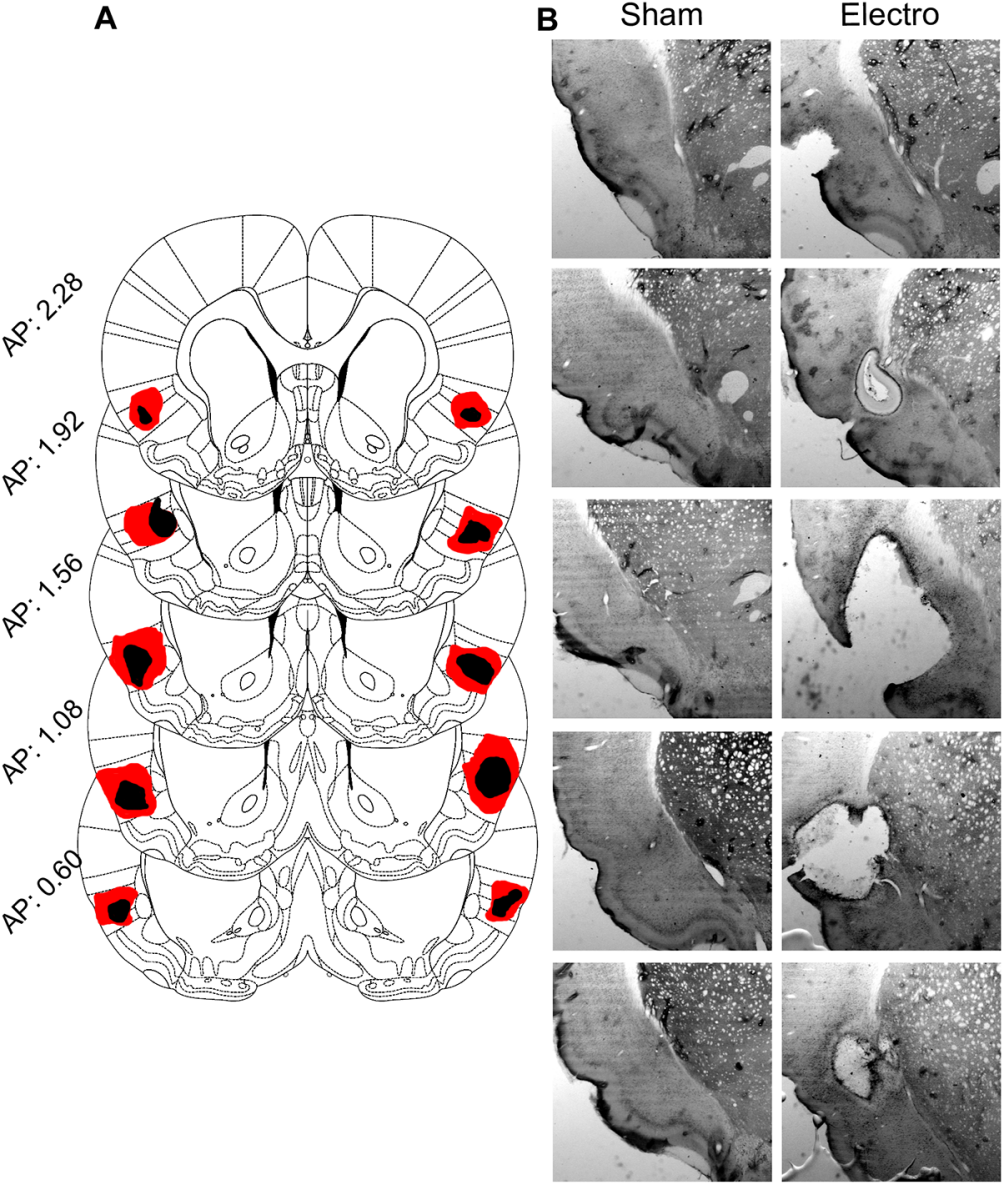
Lesion size and location. (A) Series of 5 superimposed coronal schematic representations of the largest (red) and smallest (black) lesioned areas per side found in the Electro group; numbers correspond to stereotaxic coordinates anterior to bregma. (B) Representative photomicrographs taken at 5× magnification (left column: Sham; right column: Electro) corresponding roughly to the coronal schematics seen in A.

**Fig 3:**
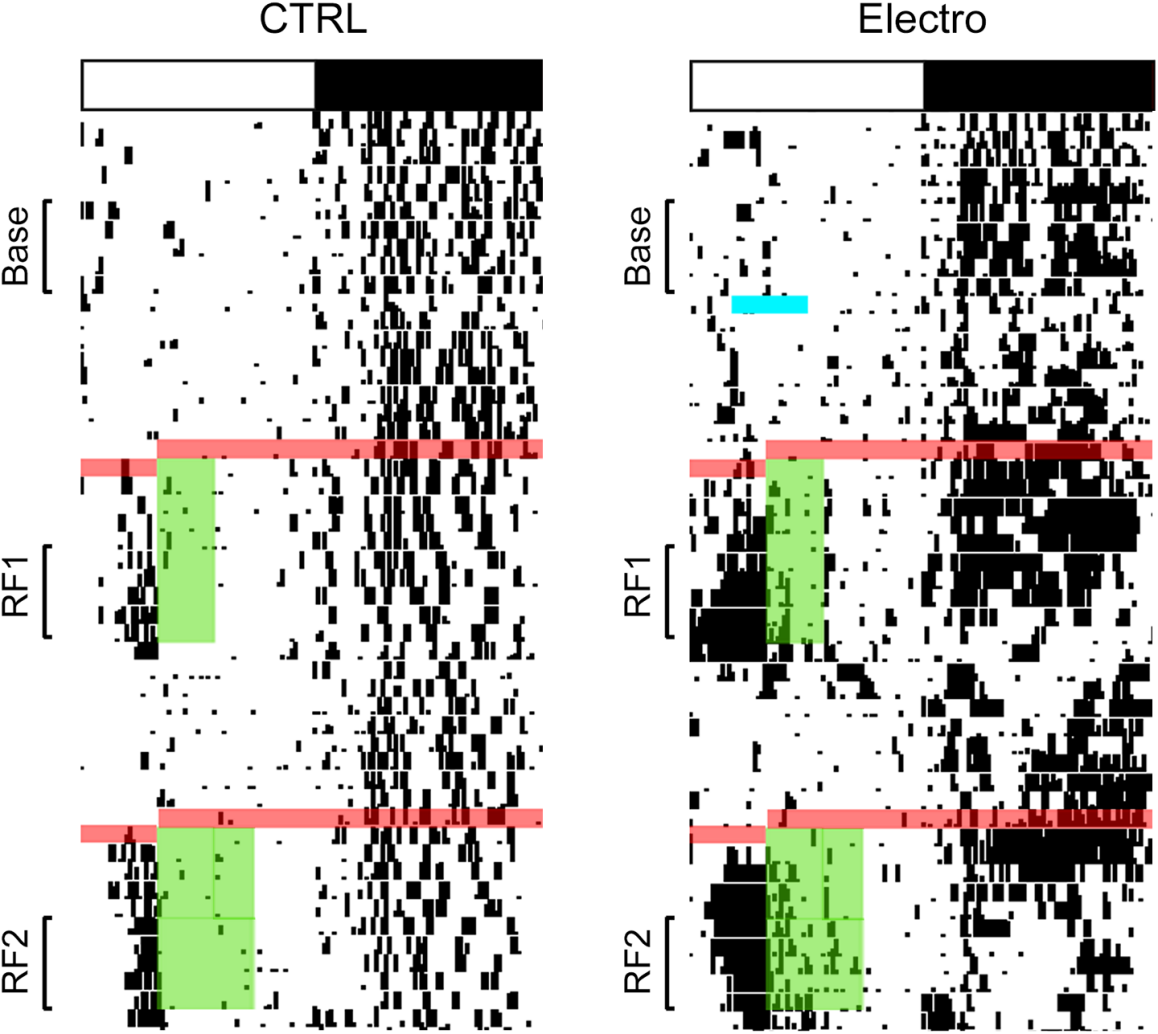
Representative actograms. White and black bar on top shows the light-dark (LD) cycle for each rat. Black marks indicate bouts of activity of at least 10 wheel revolutions/10 min. Blue line indicates the time during which the Electro-lesioned rat was out of its cage for surgery. Red lines represent the 24 h period during which rats were fasted prior to the onset of the two bouts of RF. (A) Green bars show food availability during RF1 (ZT4-ZT7) and RF2 (ZT4-ZT9). Base = 5 days of baseline; RF1 = last 5 days of the first bout of restricted feeding; RF2 = last 5 days of the second bout of restricted feeding.

**Fig 4:**
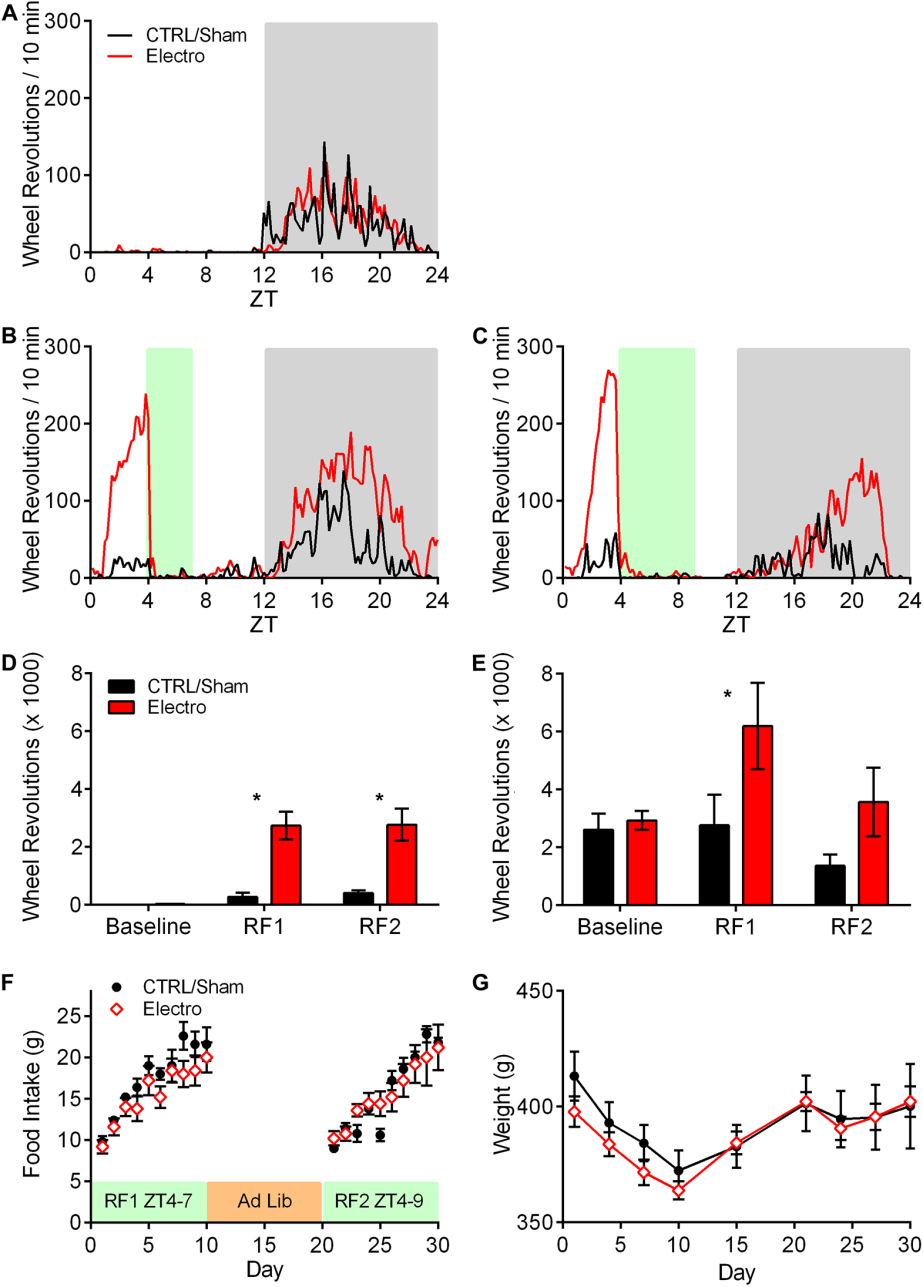
Mean and total running wheel activity. Averaged running wheel activity (over 5 days each) for CTRL/Sham (*n* = 5; black) and Electro (*n* = 5; red) during (A) Baseline, (B) RF1 (ZT4-ZT7), and (C) RF2 (ZT4-ZT9). White and grey backgrounds represent lights on and off respectively, and the green background shows food availability during RF1 and RF2. (D) Total anticipatory activity during the 3 h (ZT1-ZT4) preceding food presentation. (E) Total nighttime activity. (F) Daily food intake during RF1 and RF2 of CTRL/Sham and Electro rats. (G) Corresponding weight changes. All error bars indicate ±S.E.M. * Significantly different from CTRL/Sham. *p* < .05.

Ten rats were included in the final data analysis (CTRL/Sham, *n* = 5; Electro, *n* = 5). Schematic outlines and representative photomicrographs of Electro lesions (as well as comparable Shams) are shown in Fig 2. Single-plotted actograms showing daily wheel-running activity patterns throughout the course of the experiment are presented in Fig 3. Both groups exhibited anticipatory activity prior to food presentation during both bouts of RF. Unexpectedly, Electro rats showed larger amounts of anticipatory running during the last 5 days of RF than CTRL/Sham rats. To quantify this increase in activity, 5-day average activity graphs were plotted for each of the three experimental periods outlined in Fig 1A. Fig 4 shows the mean running wheel activity over 24 h (with a temporal resolution of 10 min) during the last 5 days of A) Baseline, B) RF1, and C) RF2. Contrary to our expectations, these findings show that bilateral electrolytic lesions of the AICa cause a significant increase, rather than a decrease, in FAA.

Total FAA (from ZT1-ZT4) and total nighttime activity were analyzed for both CTRL/Sham and Electro groups. Anticipatory activity was significantly higher in the Electro vs. CTRL/Sham group during RF1 (*p <* .001) and RF2 (*p <* .001; Fig 4D). Bonferroni post hoc tests showed a significant increase in total nighttime activity in Electro rats only during RF1 (*p <* .05; Fig 4E). No significant differences between groups existed at Baseline.

Despite showing higher FAA, Electro rats did not consume significantly more food compared to CTRL/Sham during any of the 20 days of restricted feeding (Fig 4F) or in total, although a significant main effect of time for food consumption in both groups was noted (*p* < .001), resulting from a gradual increase in the amount of food consumed each day during subsequent days of RF. No significant difference in weights was found between the two groups during the course of the experiment (Fig 4G). Body weights stabilized during the second bout of RF when rats had access to food for 5 h, instead of gradually decreasing as was seen during RF1 when food was available for only 3 h.

### Experiment 2: Length of food availability and FAA

In experiment 1, the length of food availability did not significantly alter the increase in FAA, with rats exhibiting similar levels of FAA before both 3 h and 5 h feeding windows. However, given that some studies have shown length of food availability to significantly alter the amount of FAA [33–35], food availability was adjusted to 2 h and 5 h for subsequent experiments in order to better emphasize any potential differences. Twenty-four rats were included in the final analysis (access to food for 2 h during RF1 and 5 h during RF2: CTRL/Sham 2–5, *n* = 6; Electro 2–5, *n* = 6, and access to food for 5 h during RF1 and 2 h during RF2: CTRL/Sham 5–2, *n* = 6; and Electro 5–2, *n* = 6).

**Fig 5:**
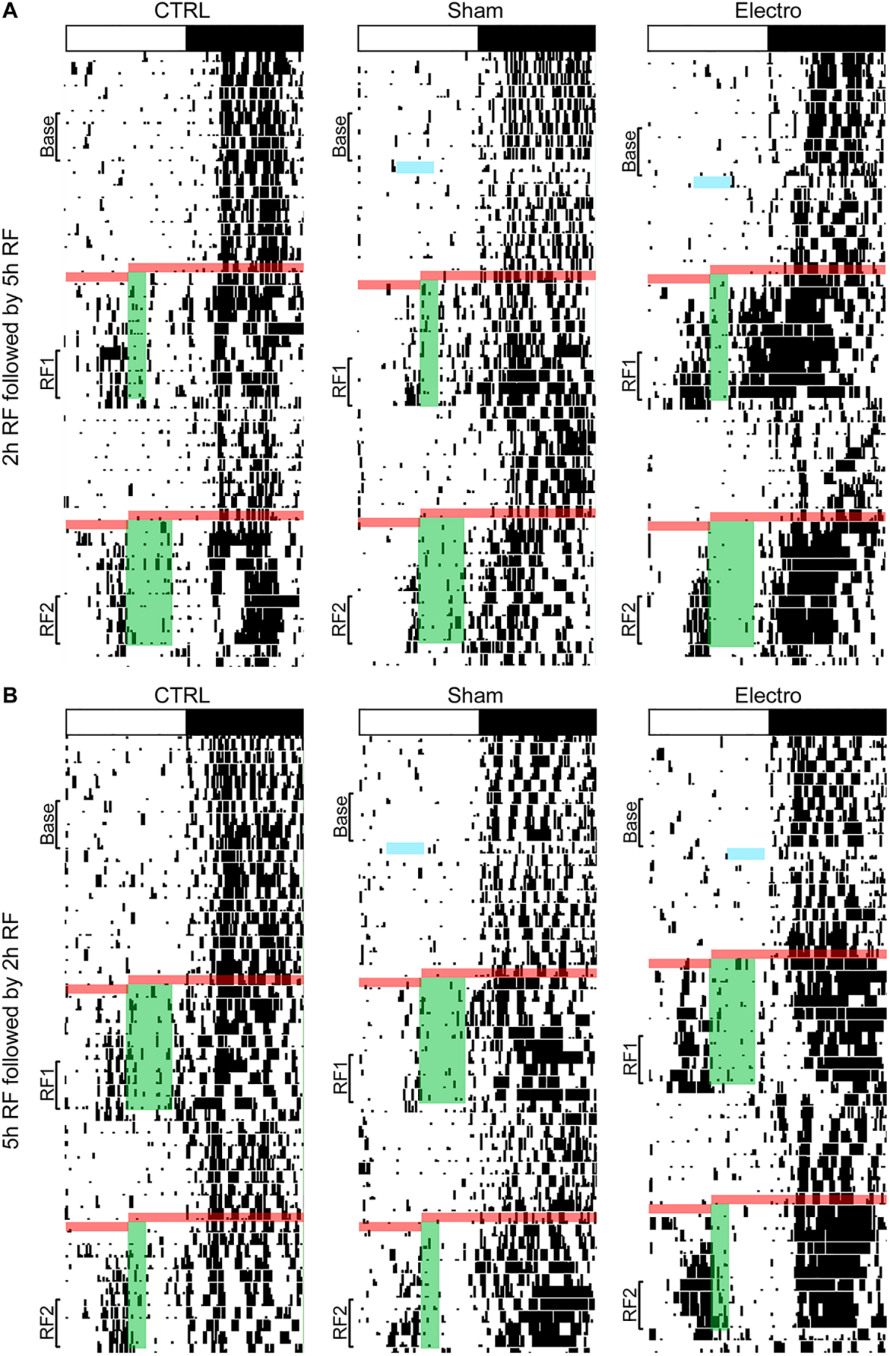
Representative actograms. White and black bar on top shows the LD cycle for each rat. Black marks indicate bouts of activity of at least 10 wheel revolutions/10 min. Arrows and blue lines indicate the time during which each rat was out of its cage for surgery. Red lines represent the 24 h period during which rats were fasted prior to the start of the two bouts of restricted feeding. Green bars depict food availability. (A) Half of the rats had access to food for 2 h (ZT6-ZT8) during RF1 for 10 days, and for 5 h (ZT6-ZT11) for 10 days during RF2, (B) the other half received the opposite order of food presentation. Base = the 5 days of baseline; RF1 = the last 5 days of the first bout of restricted feeding; RF2 = the last 5 days of the second bout of restricted feeding.

**Fig 6:**
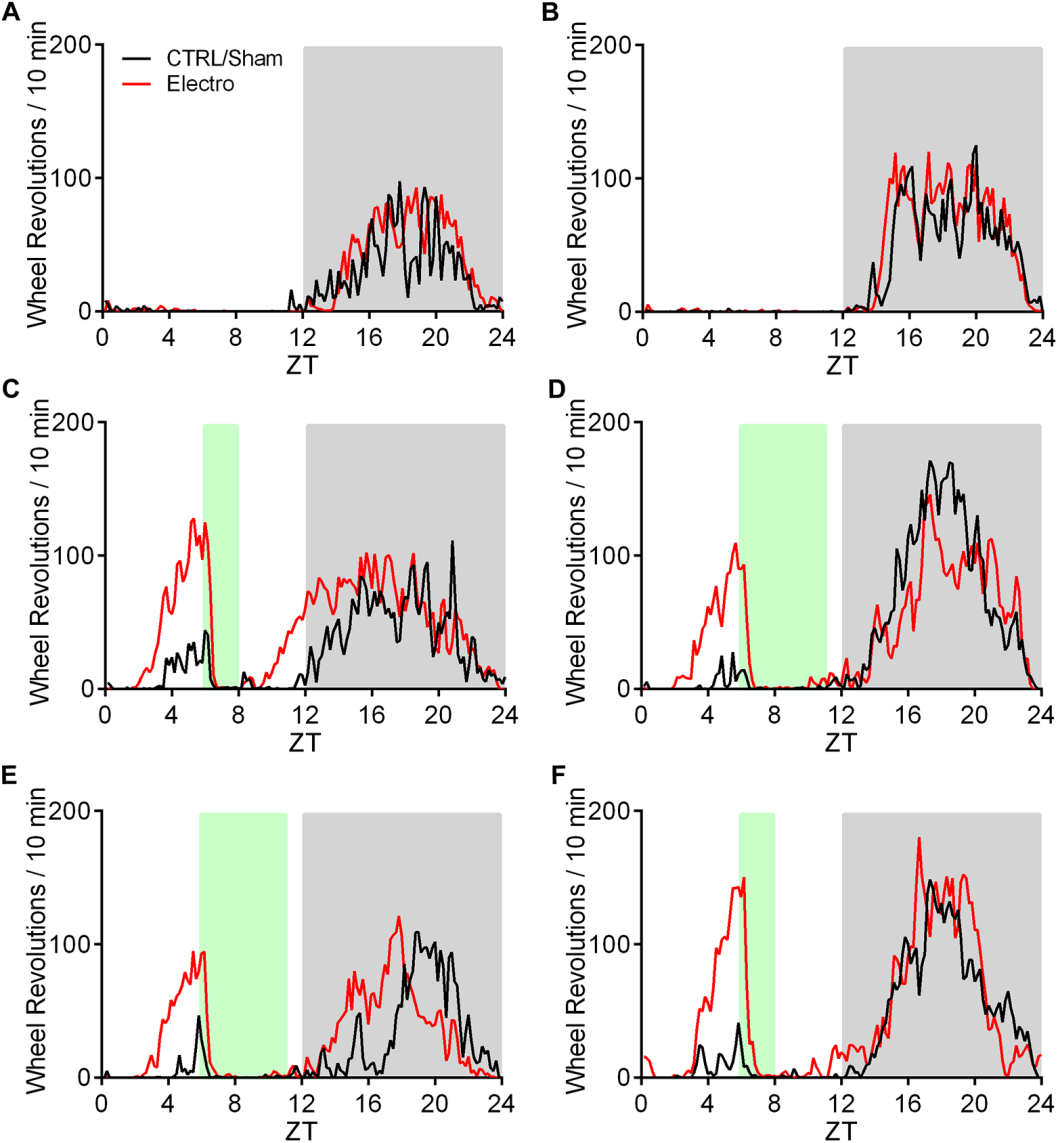
**Mean running wheel activity** over 5 days for CTRL/Sham (*n* = 6; black) and Electro (*n* = 6; red) during (A) Baseline for the 2 h—5 h groups, (C) RF1 (ZT6-ZT8), and (E) RF2 (ZT6-ZT11). (B) Baseline for the 5 h—2 h groups, (D) RF1 (ZT6-ZT11), and (F) RF2 (ZT6-ZT8). White and grey backgrounds represent lights on and off respectively, and the green background shows food availability during RF1 and RF2.

**Fig 7:**
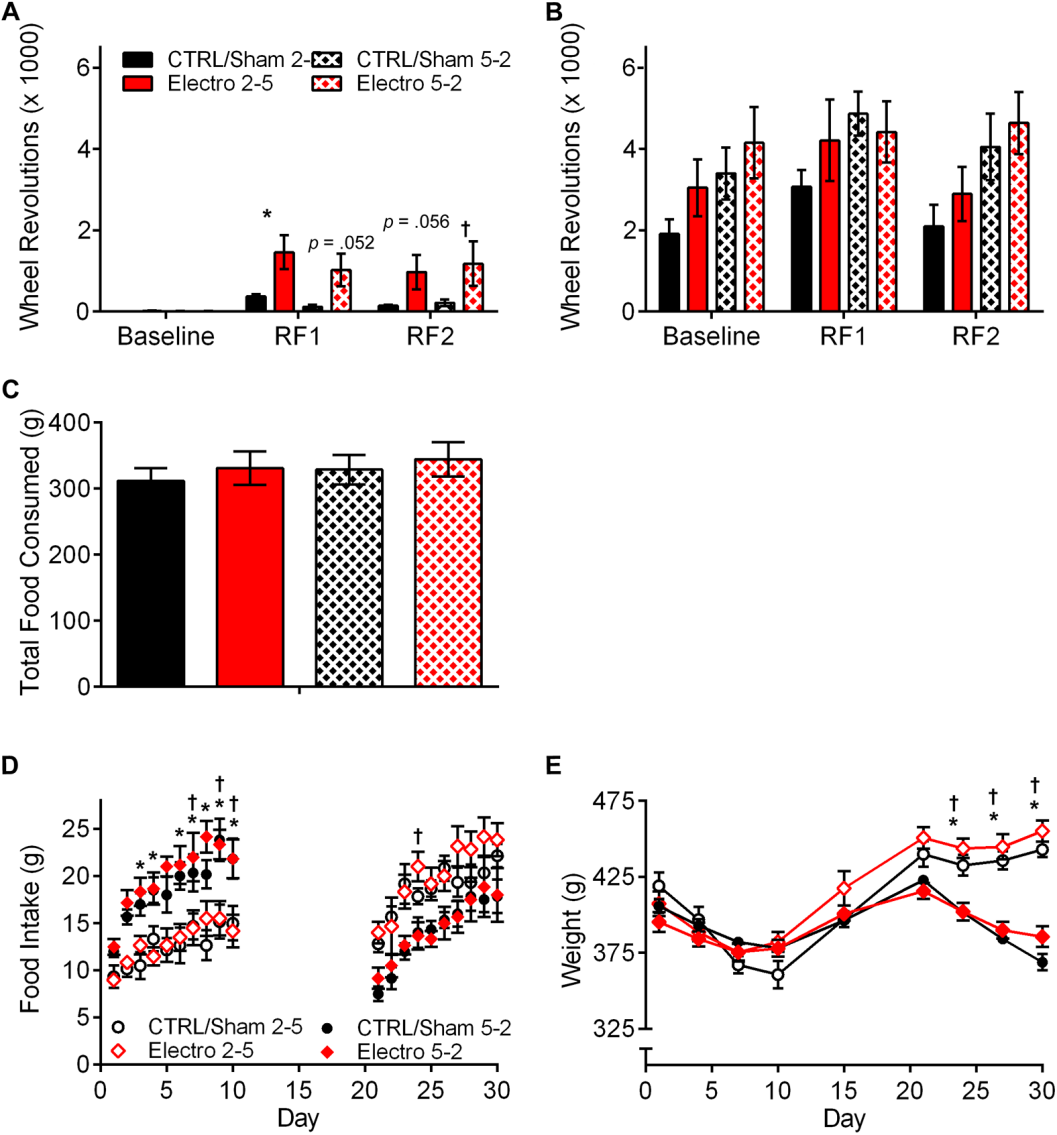
Total running wheel activity, food consumption, and weights. (A) Total anticipatory activity during the 3 h (ZT3-ZT6) preceding food presentation. (B) Total nighttime activity. (C) Total food intake during RF1 and RF2. (D) Daily food intake during RF1 and RF2. (E) Body weights over duration of experiment. All error bars indicate ±S.E.M. * Significant difference between CTRL/Sham 2–5 and Electro 2–5. **†** Significant difference between CTRL/Sham 5–2 and Electro 5–2. *p* < .05.

Wheel running activity records for representative rats from all groups are shown in Fig 5. An increase in anticipatory activity prior to food presentation is visible in all groups, with a greater increase in Electro rats. Furthermore, a larger increase in FAA can be seen prior to the 2 h food bout than the 5 h bout. Fig 6A–6F illustrates average activity graphs for the last five days of Baseline, RF1, and RF2. All rats showed negligible daytime activity and very similar nocturnal activity patterns during Baseline (Fig 6A & 6B). However, once RF commenced, Electro rats exhibited more FAA during the 3 h period (ZT3-ZT6) prior to food presentation (see Fig 7A for totals). In summary, Electro rats exhibited significantly higher FAA than CTRL/Sham rats prior to the 2 h bout of RF, regardless of whether it was their first or second exposure to an RF schedule. Specifically, anticipatory activity was significantly higher for Electro 2–5 vs. CTRL/Sham 2–5 rats during RF1 (*p* < .05) and a trend was noted for the same groups during RF2 (*p* = .056). Likewise, FAA was higher for Electro 5–2 vs. CTRL/Sham 5–2 rats during RF2 (*p* < .05) and a trend was noted during RF1 (*p* = .052). For both 5 h RF bouts, the Electro groups trended towards greater FAA compared to CTRL/Shams. Total nighttime activity levels for the four groups during Baseline, RF1, and RF2 are shown in Fig 7B. A significant main effect of time (*p* < .05) was noted indicating that rats significantly altered the amount of total nighttime activity they exhibited over the course of the experiment, yet Bonferroni post hoc tests failed to find any significant differences for specific groups.

Total food intake (Fig 7C), daily food intake during RF1 and RF2 (Fig 7D), as well as changes in average weights for the four groups are presented in Fig 7. All groups increased their food intake over the course of each experimental condition, as indicated by a significant main effect of time (*p* < .001). Duration of food presentation (2 h vs. 5 h) had an effect on daily food consumption almost exclusively during RF1, yet no effect of lesion was noted in either 2–5 or 5–2 Electro groups when compared to their respective controls. Total food consumed was not significantly different in any group (Fig 7C), yet the rats in the two 2–5 groups (Electro and CTRL/Sham) were significantly heavier than the rats in the two 5–2 groups by the end of the experiment (*p* < .001; Fig 7E).

### Experiment 3: IBO AICa lesions and treat

Experiments 1 and 2 demonstrated that large electrolytic lesions of the AICa result in a significant increase in FAA. We further investigated this effect by using a more targeted lesioning approach and by varying the palatability of food presented.

**Fig 8:**
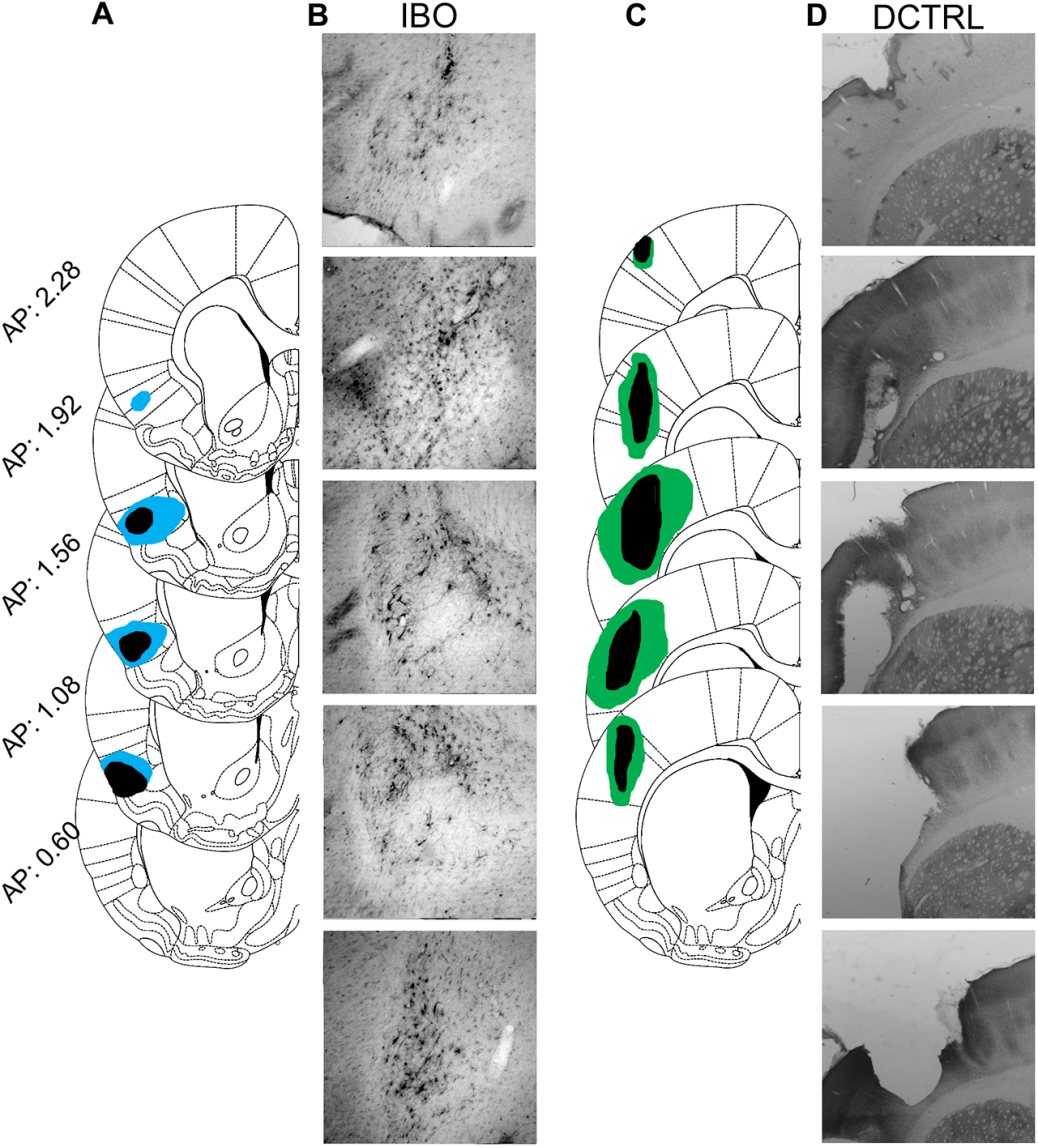
Extent of bilateral IBO and DCTRL lesions. (A) A series of 5 superimposed coronal schematic representations of the largest (blue) and smallest (black) lesioned areas found in the ibotenic acid (IBO) group; numbers correspond to stereotaxic coordinates anterior to bregma. (B) Representative photomicrographs of the lesion site taken at 10× magnification. (C) Series of 5 superimposed coronal schematic representations of the largest (green) and smallest (black) lesioned areas found in the dorsal control (DCTRL) group; numbers correspond to stereotaxic coordinates anterior to bregma. (D) Representative bilateral photomicrographs taken at 5× magnification.

**Fig 9:**
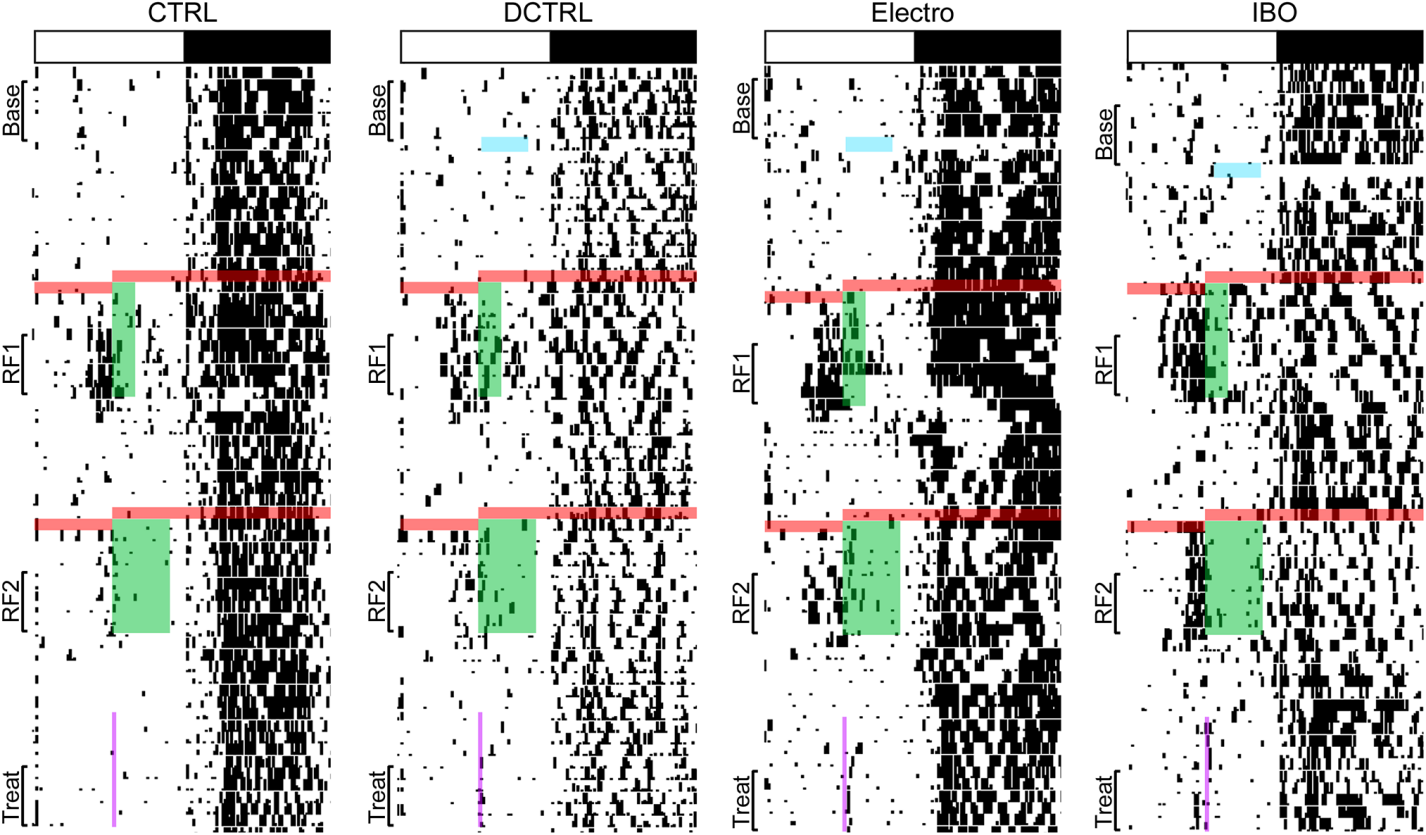
Representative single-plotted actograms depicting the course of the experiment for each condition. White and black bar on top shows the LD cycle for each rat. Black marks indicate bouts of activity of at least 10 wheel revolutions/10 min. Blue lines indicate the time during which each rat was out of its cage for surgery. Red lines highlight the 24 h period during which rats were fasted prior to the start of the two bouts of restricted feeding. Green bars represent food availability. Note that during RF1, food was given for 2 h while during RF2 it was given for 5 h. Purple vertical line at the bottom of each actogram depicts treat availability (15 min starting at ZT6).

**Fig 10:**
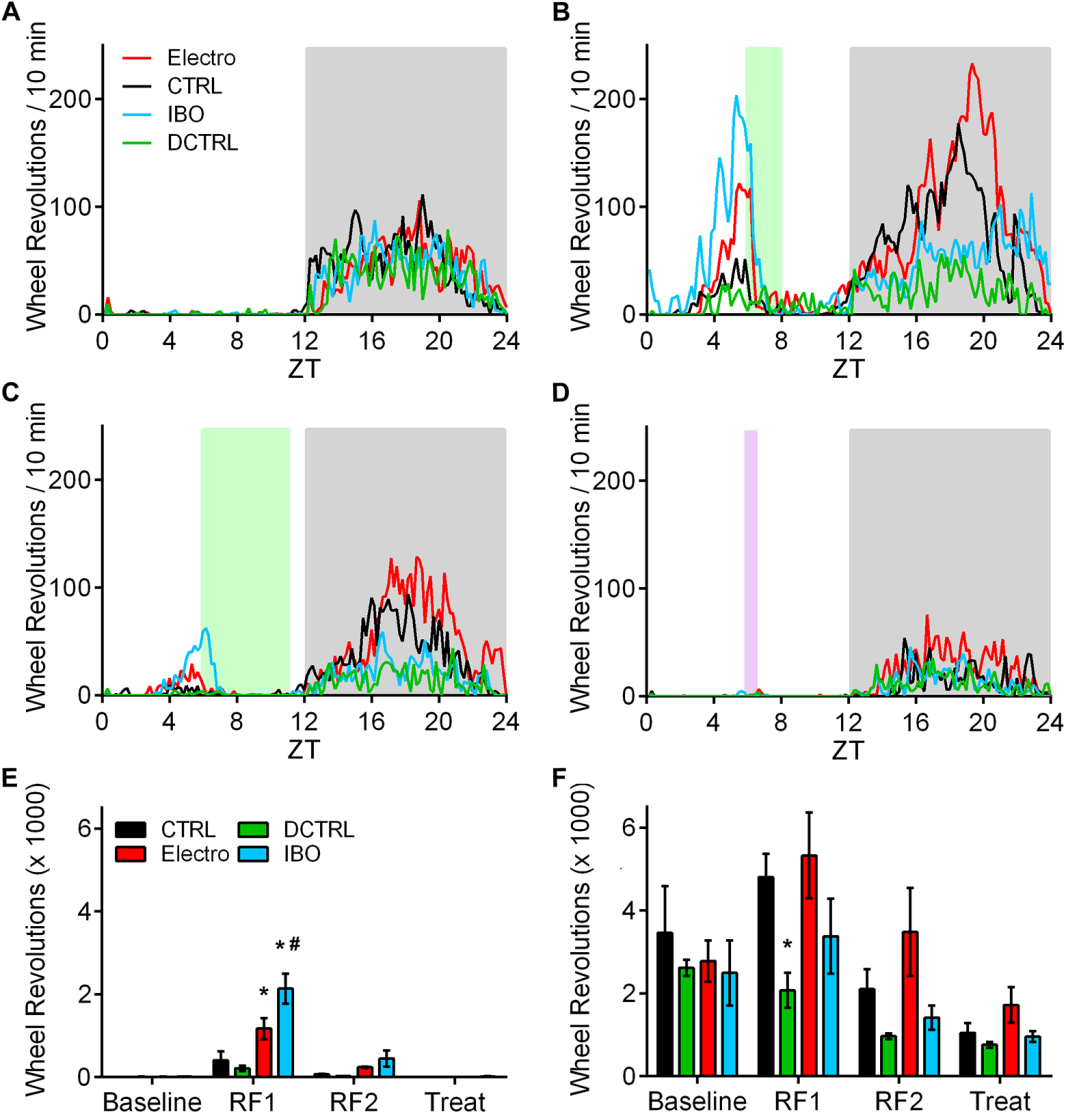
Mean activity during Baseline, RF1 (2 h), RF2 (5 h), and Treat (15 min). Averaged running wheel activity (over 5 days each) for CTRL (*n* = 5; black), DCTRL (*n* = 4; green), Electro (*n* = 5; red), and IBO (*n* = 5; blue) during (A) Baseline, (B) RF1 (ZT6-ZT8), (C) RF2 (ZT6-ZT11), and (D) Treat (starting at ZT6 for 15 min). White and grey backgrounds represent lights on and off respectively, green background shows regular rat chow availability during RF1 and RF2, and purple background depicts chocolate Ensure availability. (E) Total anticipatory activity during the 3 h (ZT3-ZT6) preceding food presentation. (F) Total nighttime activity. All error bars indicate ±S.E.M. * Significantly different from CTRL. # Significantly different from Electro. *p* < .05.

Representative photomicrographs of IBO lesions are shown in Fig 8A and the extent of DCTRL lesions in Fig 8C. Nineteen rats were included in the final data analysis (CTRL, *n* = 5; DCTRL, *n* = 4; Electro, *n* = 5; and IBO, *n* = 5). Representative single-plotted actograms are shown in Fig 9 and corresponding 5-day average activity graphs for Baseline, RF1, RF2, and Treat are shown in Fig 10A–10D. Anticipatory activity was significantly higher for Electro vs. CTRL rats (*p <* .001), and for IBO vs. CTRL (*p <* .001), during RF1 (Fig 10E). Interestingly, IBO rats showed significantly higher FAA even when compared to Electro (*p <* .001), during RF1. Only the IBO rats showed a significant increase in FAA vs. CTRL during RF2 (*p* < .05). None of the groups showed significant FAA to Treat.

All groups had similar overall activity patterns during Baseline, yet as Fig 10F shows, DCTRL rats experienced a drop in their nighttime activity levels during RF1 (*p* < .05) while still exhibiting comparable FAA to the CTRL group. This is not surprising given that bilateral electrolytic lesions to the primary sensorimotor cortex can cause some motor impairments, depending on lesion size and location [36].

Food intake and weights of all rats were monitored throughout the experiment. Despite showing significantly more FAA, Electro and IBO rats did not significantly differ in weight when compared to CTRL, nor did they consume significantly more food during most of the 20 days of restricted feeding. Lastly, Ensure consumption and weights during the 10 days of Treat were comparable between all 4 groups of rats.

### Experiment 4: Effect of IBO lesions of the AICa on open field activity

In experiment 3, we found that bilateral IBO lesions of the AICa produce a significant increase in FAA, but the effects of IBO lesions on general activity levels in novel environments had not yet been tested. Moreover, experiment 3 showed that bilateral Electro or IBO lesions of the AICa induced no FAA in response to treat, yet this was tested only after the rats had undergone two bouts of RF (2 h and 5 h respectively). Therefore, FAA prior to treat could have been affected by carry-over effects from being exposed to RF previously. In order to investigate this further, FAA prior to a 15 min treat (chocolate Ensure, starting at ZT6) given over 10 consecutive days was also ascertained in the same rats.

**Fig 11:**
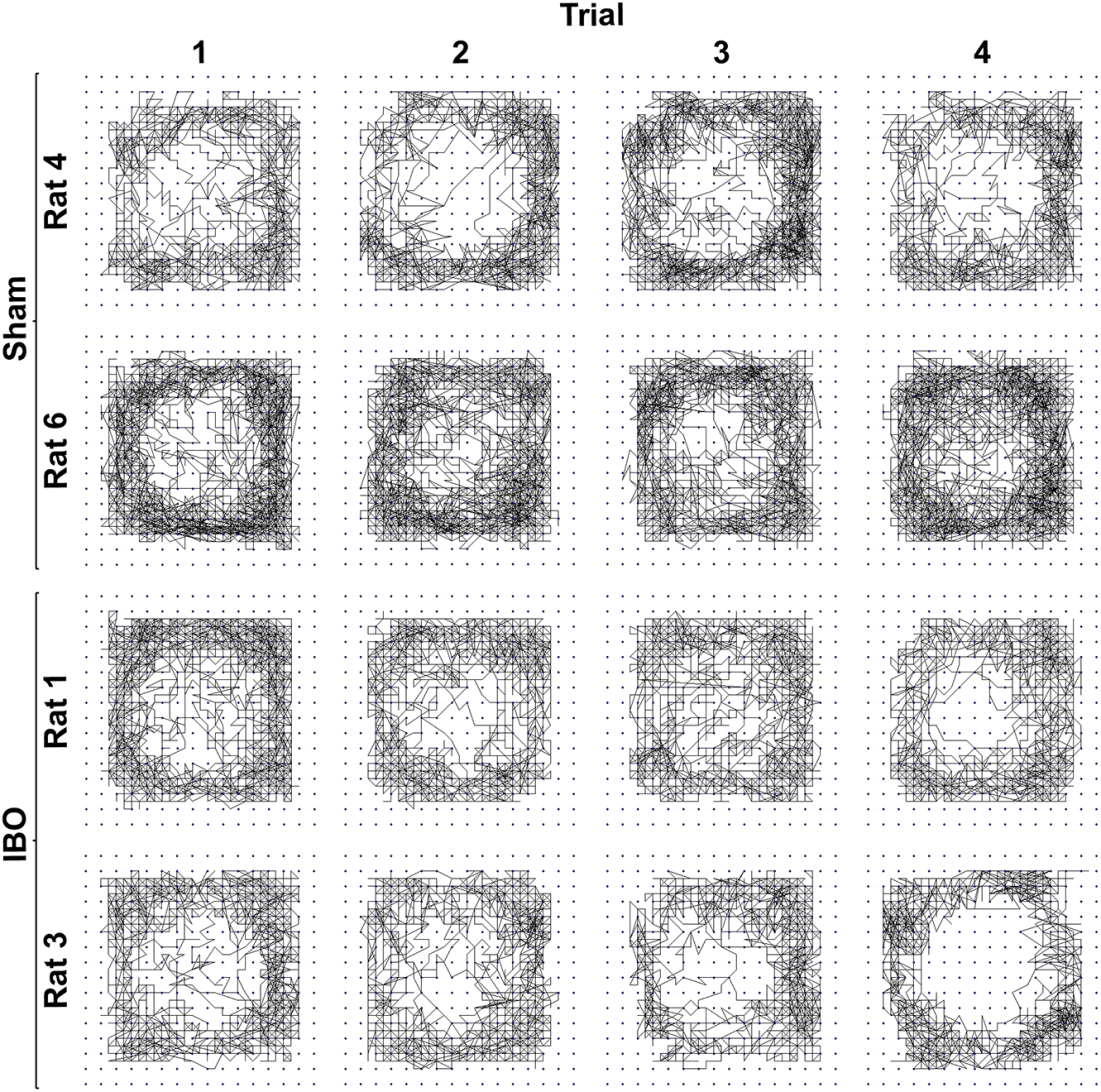
Sample movement paths during four 1 h open field trials. Individual movement paths within an open field of two rats per condition (Sham or IBO) during four consecutive 1 h trials.

To assess the effect of bilateral IBO lesions of the AICa on activity in a novel environment, the activity of eleven rats in an open field was analyzed (Sham, *n* = 6, and IBO, *n* = 5). Fig 11 depicts representative motion maps of four different rats (Sham: top two rows; IBO: bottom two rows) over the course of 1 h for four consecutive trials (refer to Fig 1D for further details). Despite some individual variability in activity levels, no significant differences were found on any activity variables analyzed between Sham and IBO rats during any 1 h trial in the open field. Planned repeated comparisons failed to reveal any significant differences between subsequent trials on any variable, suggesting that there was no effect of 24 h food deprivation (which took place before the third exposure to the open field) on total time spent moving or distance traveled.

**Fig 12:**
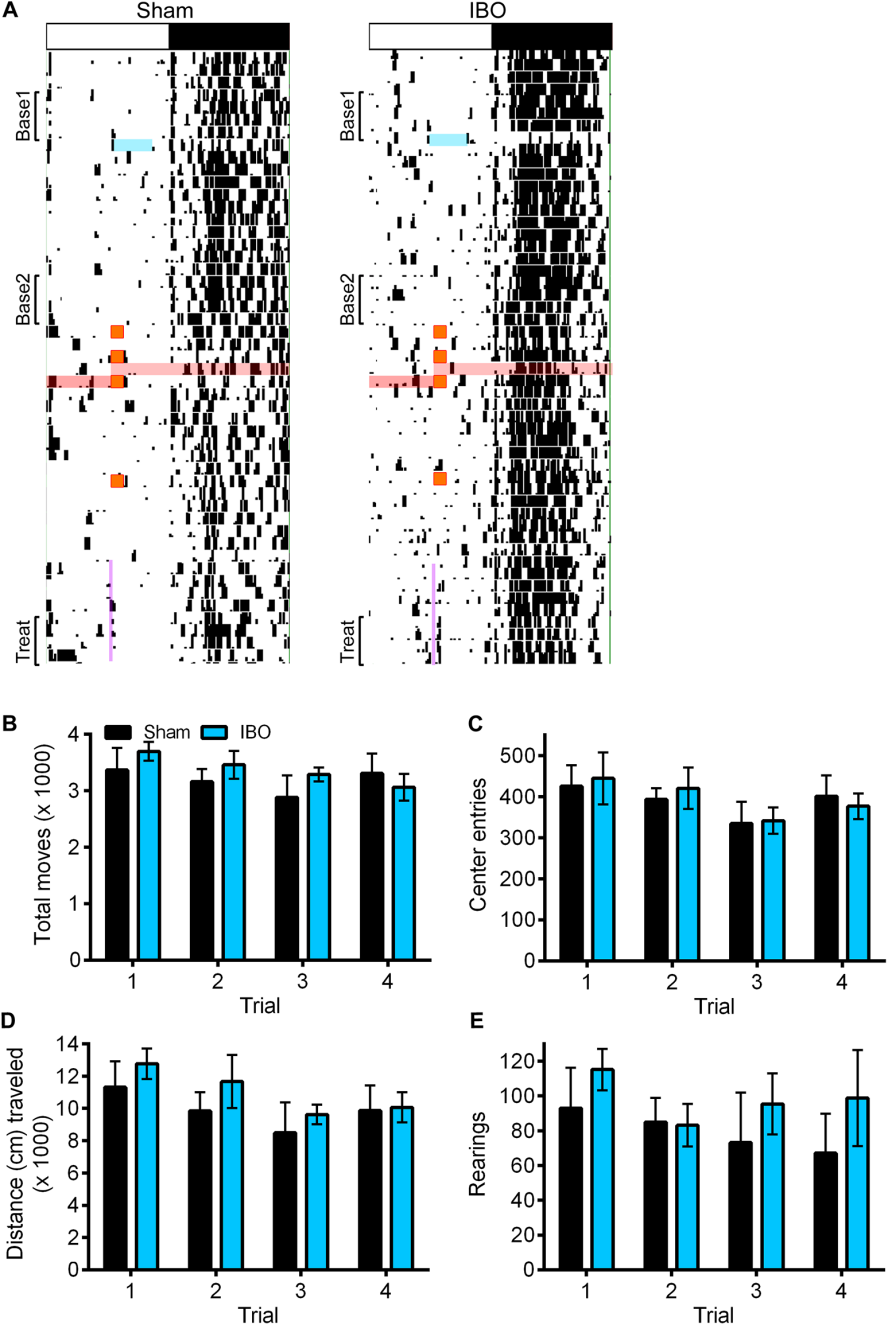
Representative actograms. White and black bar on top shows the LD cycle for each rat. Black marks indicate bouts of activity of at least 10 wheel revolutions/10 min. Blue lines highlight the time during which each rat was out of its cage for surgery. (A) Red lines illustrate the 24 h period during which rats were fasted prior to the third 1 h exposure to an open field (represented by the orange squares). Purple vertical line at the bottom of each actogram depicts chocolate Ensure availability (starting at ZT6 for 15 min). Base1 = 5 days of the initial baseline (before surgery); Base2 = last 5 days of second baseline (before treat presentation); Treat = last 5 days of treat in the presence of *ad libitum* regular rat chow. (B) Total number of moves, (C) total center entries, (D) total distance traveled, and (E) total rearings by Sham (*n* = 6; black) and IBO (*n* = 5; blue) rats during 1 h (ZT6-ZT7) in an open field over four consecutive trials. All error bars indicate ±S.E.M. *p* < .05.

Representative single-plotted actograms of wheel running activity during daily Treat delivery are shown in Fig 12A. Neither group showed significant anticipation to Treat, nor were there any significant differences in total nighttime activity between IBO and Sham rats during any portion of the experiment found. We examined daily wheel running patterns for any effect of exposure to the open field. The nocturnal running wheel activity of all rats decreased significantly (*p* < .001) following the first 1 h exposure to the open field (ZT6-ZT7). This drop in activity levels persisted for the remainder of the experiment. Subsequent re-exposures to the open field did not have any additional effects on running wheel activity. There were no significant differences between IBO and Sham rats on nighttime activity. Nonetheless, due to the reduction in running wheel activity, two baselines were considered for making statistical comparisons: Baseline1 spanned the last 5 days prior to surgery, and Baseline2 spanned the last 5 days prior to Treat (see Fig 1D for detailed outline).

## Discussion

Rats with bilateral electrolytic or cytotoxic lesions of the AICa showed significantly more FAA during the 3 h preceding food presentation. This effect was very robust prior to 2-h and 3-h meals but showed some variability prior to 5-h meals. Despite showing higher FAA, experimental rats did not consume significantly more food than the various control groups throughout each experiment.

These findings suggest that the AICa is a brain region whose effect on FAA is in part dependent on meal duration. It has long been known that meal duration affects the amount of food consumed [33]. Shorter meal duration results in a higher value placed on each calorie consumed given that food is available for less time [37], increasing the motivation for food and food seeking in the future. Several studies have shown the NAc [38] and amygdala [39] to be activated following exposure to food. Exposure to food was also found to activate the insula in food-deprived subjects [40]. Given the AICa's role in influencing motivated behaviours through integrating visceral sensations and emotional states [20] via its bi-directional projections to limbic structures such as the NAc and amygdala [23], the AICa may be more strongly activated in response to a 3 h than a 5 h meal.

There are multiple compensatory mechanisms which may diminish the impact of bilateral AICa lesions. One such mechanism could be in part made up by the dopaminergic projections of the mesocorticolimbic pathway, which reach the NAc without passing through the AICa, thus sending signals regarding the rewarding nature of a meal directly to the NAc through other relays than the AICa from the parabrachial nucleus. Despite showing higher FAA, experimental rats with bilateral lesions did not consume significantly more food than the various control groups. However, a significant main effect of time on food consumption was found in all groups. This finding makes intuitive sense given that rats began each RF period with sporadic food consumption before feeding became robust, leading to a significant increase in their daily food intake over time.

Interestingly, large bilateral radiofrequency ablations of the NAc, have been shown to not disrupt FAA [12] yet more selective NMDA excitotoxic lesions of the NAc shell showed a significant increase in FAA while selective NMDA excitotoxic lesions of the NAc core produced a substantial decearse in FAA [41]. Taken together, these data highlight the variance in responses one can attain by choosing one type of lesion over another, as well as opting for a more targeted lesion versus a more general lesion impacting multiple brain regions. The suppression of the NAc shell and core can have opposite effects on similar behavioural outputs due to their specific afferent and efferent projections but especially due to the differential concentration of D1-type and D2-type dopamine receptors within each sub-region of the NAc. As such, it is not entirely surprising that lesioning the entire NAc together with other adjacent dorsal areas may negate the more specific effects that can be noted when lesioning either NAc sub-division on its own with a more targeted lesioning approach. Given that the AICa is interconnected with both the NAc core and shell to slightly different degrees, future studies should use excitotoxic lesions such as IBO of the AICa and measures of cell activity such as cFos immunoreactivity can be used in order to better assess the impact that IBO lesions of the AICa may have on neural activity within the NAc and on FAA in general.

In the present series of experiments, IBO lesions led to the largest increases in FAA suggesting that fibers passing through the AICa can modulate behaviour further, even with the AICa lesioned. A daily treat failed to elicit a significant increase in FAA in any of the four groups suggesting that the palatability of food plays a lesser role than the alleviation of a negative metabolic state resulting from food deprivation [27, 42]. Because IBO lesions resulted in a significantly larger increase in FAA than electrolytic lesions, it suggests that the AICa itself provides some kind of inhibitory regulation over FAA, and that fibers of passage through the AICa may play a positive modulatory role in increasing FAA. Experiment 4 found that bilateral IBO lesions to the AICa lead to a selective increase in FAA only under an RF paradigm, and that a palatable treat fails to produce similar levels of anticipation. Moreover, IBO rats did not differ significantly from Shams on any of the measures used in the open field, indicating that lesions to the AICa do not alter general levels of locomotor activity. One puzzling finding, however, was the significant reduction in running wheel activity following the first exposure to the open field. Further study is necessary in order to determine whether this is a consistent result or whether it was due to other variables not accounted for in the present experimental design.

Of the 24 rats excluded from the various experiments for unsuccessful surgery, 14 had unilateral lesions of the AICa. Four of these rats showed a slight increase in FAA compared to CTRL/Sham but this increase was not significant. Although the degree of FAA in all 14 rats varied considerably, the increase in FAA in several of these rats nonetheless suggests the loss of activity in the AICa of only one hemisphere may be sufficient to disinhibit FAA to meal presentation.

Analysis of wheel running activity indicated that experimental rats showed significant increases in FAA prior to all 2 h meals, regardless of whether the 2 h meal was presented first or followed a 5 h meal during RF1. While no significant effects of order of food presentation on FAA were found, there were carry-over effects on sequential food consumption and weight gain. Specifically, rats that had access to 2 h meals during RF1 followed by 5 h meals during RF2 were significantly heavier by the end of the experiment, despite not consuming more food than the other groups. While these latter findings may appear less germane to the scope of this study, they are in fact relevant because they can help to further elucidate the role played by varying RF schedules on total weight gain and have important implications for metabolic function. This in turn can assist in better interpreting other findings, such as discrepancies in weight gain under varying conditions and time exposures to food. However, given that no significant differences between the CTRL/Sham groups and Electro groups were observed, the AICa is likely not the main brain region involved in this effect.

In summary, the present study found that bilateral lesions to the AICa significantly increase FAA, especially preceding instances of brief access to food such as a daily 2 h meal. These findings suggest that this brain region, already known to play a role in receiving interoceptive signals from the body and influencing motivated behaviours in response to these signals, is an important component of the broader network of brain regions involved in producing FAA.
